# Identification of Marine Compounds Inhibiting NF-κBInducing Kinase Through Molecular Docking and Molecular Dynamics Simulations

**DOI:** 10.3390/biom14121490

**Published:** 2024-11-22

**Authors:** Muhammad Yasir, Jinyoung Park, Eun-Taek Han, Jin-Hee Han, Won Sun Park, Jongseon Choe, Wanjoo Chun

**Affiliations:** 1 Department of Pharmacology, Kangwon National University School of Medicine, Chuncheon 24341, Republic of Korea; yasir.khokhar1999@gmail.com (M.Y.); jinyoung0326@kangwon.ac.kr (J.P.); 2 Department of Medical Environmental Biology and Tropical Medicine, Kangwon National University School of Medicine, Chuncheon 24341, Republic of Korea; ethan@kangwon.ac.kr (E.-T.H.); han.han@kangwon.ac.kr (J.-H.H.); 3 Department of Physiology, Kangwon National University School of Medicine, Chuncheon 24341, Republic of Korea; parkws@kangwon.ac.kr; 4 Department of Microbiology and Immunology, Kangwon National University School of Medicine, Chuncheon 24341, Republic of Korea; jchoe@kangwon.ac.kr

**Keywords:** NF-κB-inducing kinase (NIK) inhibition, marine-sourced compounds, molecular docking, molecular dynamics simulation, free energy calculation

## Abstract

NF-κB-inducing kinase (NIK) plays a pivotal role in regulating both the canonical and non-canonical NF-κB signaling pathways, driving the expression of proteins involved in inflammation, immune responses, and cell survival. Overactivation of NIK is linked to various pathological conditions, including chronic inflammation, autoimmune diseases, metabolic disorders, and cancer progression. As such, NIK represents a compelling target for therapeutic intervention in these diseases. In this study, we explored the inhibitory potential of marine-derived compounds against NIK using integrated computational techniques, including molecular docking, molecular dynamics (MD) simulations, and free energy calculations. By screening a library of bioactive marine compounds, we identified several promising candidates with strong binding affinity to the NIK active site. By continuously narrowing down the library at each step, we found that the compounds santacruzamate A, xanthosine, and actinonine stand out at each step by demonstrating compact binding, highly stable interactions, and the most favorable free energy profiles, indicating their potential as effective NIK inhibitors. These findings not only advance our understanding of marine compounds as valuable resources for drug discovery but also highlight their potential for the development of natural anti-inflammatory therapies targeting NIK. This study opens new avenues for future research and therapeutic development aimed at combating inflammation and cancer through NIK inhibition.

## 1. Introduction

NF-κB-inducing kinase (NIK), also known as MAP3K14, plays a crucial role in regulating the noncanonical NF-κB signaling pathway, which is fundamental to a wide range of physiological processes, including immune responses and cell survival [1,2]. NIK is activated following the binding of ligands to members of the tumor necrosis factor (TNF) receptor family, such as BAFFR, CD40, RANKL, and TWEAK [3]. Under normal conditions, NIK is kept inactive by being part of a complex with TRAF3, TRAF2, and cellular inhibitors of apoptosis proteins (cIAP1/2), which leads to its continuous degradation. Upon ligand binding to these receptors, TRAF3 is degraded, releasing NIK from the complex. Once released, NIK becomes stabilized and activated, phosphorylating the Ser176 residue of IKKα, leading to further downstream signaling [4].

The activated NIK-IKKα complex then phosphorylates p100, which undergoes proteolytic cleavage to form p52 [5]. The p52 protein, together with RELB, forms a heterodimer that translocates to the nucleus, where it promotes the transcription of target genes involved in immune and inflammatory responses [6,7]. This pathway plays a pivotal role in maintaining immune homeostasis, but its dysregulation has been implicated in various pathological conditions. Aberrant NIK activation is associated with heightened NF-κB2 activity, which contributes to the development and progression of several diseases, including autoimmune disorders, inflammatory diseases, and various forms of cancer [4,8]. This makes NIK a promising therapeutic target for inhibiting pathogenic NF-κB signaling.

In particular, the stabilization and abnormal activation of NIK have been linked to inflammatory diseases [9,10]. Given the role of NIK in promoting pro-inflammatory gene expression and supporting tumor cell survival, the inhibition of NIK has become a significant focus for the development of therapeutic strategies aimed at mitigating these harmful effects. By blocking NIK’s function, it is possible to suppress the overactive NF-κB signaling, thereby reducing inflammation and potentially limiting tumor progression.

In cancers, the exploitation of the noncanonical NF-κB pathway happens to resist apoptosis and promote an immunosuppressive tumor microenvironment, aiding tumor progression and treatment resistance [11,12,13]. Likewise, in autoimmune and inflammatory diseases, NIK-driven NF-κB activity leads to the production of pro-inflammatory cytokines and chemokines, exacerbating the disease state [14,15,16]. Targeting NIK has thus emerged as an attractive therapeutic strategy for reducing inflammation and altering the tumor microenvironment, offering a pathway to alleviate both inflammatory diseases and cancer.

This study focused on identifying potential inhibitors of NIK using marine-sourced compounds. Marine organisms have proven to be a valuable source of unique small molecules with diverse chemical structures and powerful biological activities; exploring their binding affinity and stability through molecular docking and molecular dynamics simulations could pave the way for a number of investigational studies. The computational methods employed here, including molecular docking analysis, molecular dynamics, and free energy calculations, provide insights into the interaction mechanisms of these compounds with NIK. The goal was to discover compounds that can effectively suppress NIK activation, thereby offering therapeutic potential for treating both inflammatory diseases and NIK-related cancers. We ranked those compounds to highlight their potential as NIK inhibitors.

## 2. Materials and Methods

### 2.1. NIK Structure Retrieval and Identification of Active Binding Sites

The structural data for human NIK, characterized by the presence of alpha-helices, beta-sheets, coils, and turns, was sourced from the Protein Data Bank (PDB ID: 4IDT) at a resolution of 2.40 Å. This three-dimensional model underwent a comprehensive quantitative structural analysis through the VADAR online tool (http://vadar.wishartlab.com/) (accessed on 25 September 2024) [17]. Subsequently, the protein structure was subjected to energy minimization and a detailed Ramachandran plot analysis, utilizing the UCSF Chimera v1.16 and Discovery Studio Client v22 software packages [18,19], respectively, for the structural integrity and stability analysis of the human NIK protein.

The complex consisting of NIK and its crystal-bound ligand, retrieved from the Protein Data Bank (PDB), was further subjected to active site identification. Utilizing Discovery Studio’s ligand interaction method, the interacting amino acids were meticulously identified to ensure the accurate generation of the binding site. Following this, the bound ligand was selected, and a binding sphere was established using the Define Binding Site window within Discovery Studio. To further improve the accuracy of the docking process, the binding sphere was refined by applying specific constraints to the identified amino acids. This meticulous approach ensured a precise and reliable analysis of the binding interactions.

### 2.2. Ligand Preparation

Marine organisms have been recognized as a rich source of distinct small molecules, exhibiting a wide array of chemical structures and potent biological activities. The primary focus of our investigation was to assess the inhibitory potential of marine-derived natural products specifically targeting NIK. A specialized collection of marine-derived natural compounds, comprising 43 unique substances, was obtained from the online vendor MedChemExpress (MCE) (https://www.medchemexpress.com/screening/marine-sourced-natural-product-library.html) (accessed on 19 September 2024) for the purpose of screening. This unique library comprises compounds known for their diverse biological activities, with many showing promise in anti-inflammatory and anticancer applications. We selected this database due to the novelty and therapeutic potential of marine compounds, an area that remains less explored for targeting the NF-κB pathway, particularly NIK. Therefore, the library was accessed and subjected to further optimization using the Discovery Studio Client v22 and UCSF Chimera v1.16 software tools. This process aimed to enhance the potential of these marine-sourced compounds for various applications.

### 2.3. Molecular Docking

Molecular docking serves as a prevalent technique for assessing the interactions between ligands and receptors. This method employs scoring algorithms to predict the binding strength or negative energy score of protein–ligand complexes by examining their preferred orientations. This predictive approach provides valuable insights into the stability and strength of these interactions [20,21,22,23]. The protein was prepared by eliminating the pre-bound ligand and water molecules, followed by the addition of hydrogen atoms, a process facilitated by Discovery Studio’s receptor preparation module. Furthermore, the candidate compounds underwent ligand preparation, which included the generation of tautomers, adjustment of ionization states, and correction of any valence issues, utilizing the Ligand Preparation module in Discovery Studio. Molecular docking of these ligands against the target protein, NIK, was conducted using the CDocker module in Discovery Studio, employing default orientation and conformation settings. The evaluation of the best-docked complexes was based on the lowest docking energy values, quantified in kcal/mol. This meticulous approach ensured a thorough and accurate assessment of the protein–ligand interactions.

### 2.4. Molecular Dynamics Simulations

The top 10 compounds exhibiting the lowest docking energy were chosen for a 100-nanosecond (ns) Molecular Dynamics (MD) simulation. The protocols for this MD simulation experiment were derived from our previously published research papers. This approach ensured a consistent and reliable methodology for the simulation process [24,25]. Therefore, The CHARMM36 force field was configured using the solution builder protocol on the CHARMM-GUI server (https://www.charmm-gui.org/?doc=input/solution) (accessed on 10 October 2024). This platform was also utilized to create input files necessary for MD simulations with GROMACS v2019.3, ensuring the accurate and efficient setup of the force field and simulation parameters [26].

The system was solvated employing the TIP3P-3 point water model within a cubic box, applying periodic boundary conditions. Neutralization was attained by introducing counter ions. The electrostatic and van der Waals interactions were computed using the Verlet method, with a cut-off radius of 10 Å, and the LINCS algorithm was utilized to constrain bond lengths during the simulations. Furthermore, the Particle Mesh Ewald (PME) approach was employed for accurate computation of electrostatic interactions. The solvated systems were prepared using the steepest descent energy minimization method. The system underwent two equilibration phases: initially under constant temperature and constant volume (NVT) conditions, followed by constant temperature and constant pressure (NPT) conditions. A built-in Python script from CHARMM-GUI was utilized to convert the GROMACS topology (top) and parameter (itp) files for MD simulations. The structural analysis of the protein–ligand complexes was conducted using GROMACS v2019.3 on a Linux platform. This comprehensive approach ensured a thorough and reliable analysis of the molecular dynamics simulations [27]. Therefore, a time step of 2 femtoseconds (fs) was utilized to execute the Molecular Dynamics (MD) simulations in GROMACS v2019.3. This choice of time step ensured accurate and stable simulation trajectories, allowing for the detailed analysis of protein–ligand interactions over time.

Furthermore, the RMSD, hydrogen bonds, and MD interaction energies were calculated by analyzing the protein–ligand interactions throughout the MD simulation trajectory, focusing on the overall stability of ligands, van der Waals forces, electrostatic interactions, and hydrogen bonding interactions between the compound and the active site of NIK.

### 2.5. gmxMMPBSA Binding Free Energy Calculation

A widely used free energy calculation tool named gmx_MMPBSA v1.6.3 was developed to calculate the end-state free energies of protein–ligand complexes using MD trajectory data generated by GROMACS v2019.3. This program facilitates the analysis of free energy changes, providing insights into the stability and binding affinity of the complexes [28]. Binding free energy predictions were performed using the MM/PBSA approach, which involves analyzing MD simulation trajectories in explicit solvent. This method evaluates the three components complex, receptor, and ligand separately to provide insights into the binding affinity and stability of the protein–ligand interactions [29]. The binding free energy (ΔG_binding_) of the lead compounds in complex with the protein was calculated using the following equation:

ΔG_binding_ = G_complex_ − (G_protein_ + G_ligand_) (1)

This equation represents the difference in free energy between the complex (G_complex_) and the sum of the free energies of the individual receptor (G_receptor_) and ligand (G_ligand_). This approach allows for a quantitative assessment of the binding affinity between the lead compounds and the protein.

## 3. Results and Discussion

### 3.1. Structure and Binding Site Analysis

**Figure 1:**
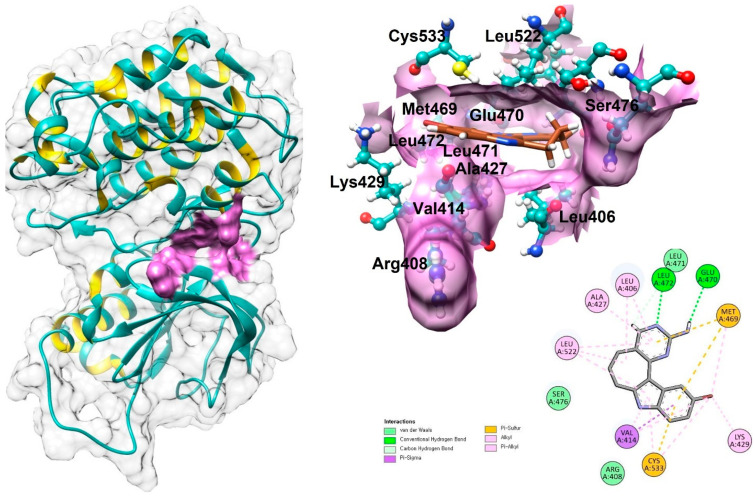
The positioning of the active binding pocket in the 3D structure of NIK and the interacting amino acid residues with the co-crystalized ligand.

The NIK protein consists of a single chain composed of 356 amino acids. Its three-dimensional structure, obtained from the Protein Data Bank (PDB ID: 4IDT), has a resolution of 2.40 Å. The protein’s architecture includes α-helices, β-sheets, and coils (Figure 1).

VADAR 1.8 statistical analysis indicates that the protein comprises approximately 33% α-helices, 23% β-sheets, 43% coils, and 27% turns. Ramachandran plots show that 96.4% of the residues are located in favored regions, 99.7% are in allowed regions, with a single outlier (Trp665) for the dihedral angles phi (φ) and psi (ψ) (Figure 1).

Moreover, the functionality of a binding pocket is determined by its shape, position within the protein, and the arrangement of surrounding amino acid residues which play an important part in the interaction [30,31,32]. Using Discovery Studio’s ligand interaction method, the binding pocket residues of NIK were identified as Cys533, Lue522, Ser476, Met469, Glu470, Leu472, Leu471, Ala427, Lys429, Val414, Leu406, and Arg408. These residues were further validated against existing published data [33]. To investigate the accurate interaction of marine compounds within the active site of NIK, the binding sphere coordinates were set to X = 97.4430, Y = 46.3800, and Z = 120.1386, with a radius of 7.3028, based on the binding pocket residues (Figure 1).

### 3.2. Marine Library Accession and Optimization

Marine compounds have great potential for pharmaceutical applications, with several already approved as drugs. The marine environment has yielded numerous pharmacologically active substances, demonstrating its importance in drug discovery [34].

One notable example is ziconotide, a peptide derived from the venom of a tropical cone snail. This compound made history in December 2004, when it became the first marine-derived drug approved in the United States for pain management [35]. Following this breakthrough, trabectedin achieved another milestone in October 2007 as the first marine-sourced anticancer drug to receive approval in the European Union [36].

The NIK pathway has been extensively studied for its role in various diseases. However, there is still a significant gap in research regarding the potential of marine-sourced compounds in targeting this pathway, particularly for inflammatory conditions [37]. Therefore, a unique library of marine-sourced natural compounds was accessed from an online vendor’s website, MCE (MedChemExpress mailto: https://www.medchemexpress.com/screening/marine-sourced-natural-product-library.html) (accessed on 19 September 2024), containing a group of 43 compounds for the screening process.

### 3.3. Molecular Docking

The CDocker module of the Discovery Studio predicts the binding strength and interactions of small molecules and a target protein by calculating two primary energy values: CDocker energy and CDocker interaction energy. Three compounds were rejected in the molecular docking ligand preparation process. However, the full molecular docking results are presented in Supplementary Data Table S1.

CDocker energy (kcal/mol) represents the internal and overall energy of the ligand and protein complex. A lower CDocker energy score generally indicates a more stable and favorable binding conformation, implying stronger interactions between the compound and the target. CDocker interaction energy (kcal/mol), on the other hand, focuses on the direct interaction energy between the ligand and the protein (Table 1). High negative interaction energy suggests that the compound forms more favorable hydrogen bonds or hydrophobic interactions with the active site residues of NIK.

Among the compounds docked, santacruzamate A shows the most favorable CDocker energy at −49.0692 kcal/mol, suggesting a highly stable binding conformation with NIK. Actinonine, with a CDocker energy of −45.5211 kcal/mol, also demonstrates a strong binding affinity. Its CDocker interaction energy (−52.1040 kcal/mol) further supports its strong binding potential. Furthermore, Cosbiol ranks third, with a CDocker energy of −34.8735 kcal/mol. It reflects stable interaction with NIK as its interaction energy of −49.9577 kcal/mol suggests stronger binding interactions than its overall docking energy.

Lumichrome and m3-indolylacetate also show CDocker energies of −31.5584 kcal/mol and −26.8642 kcal/mol, respectively, indicating good binding strength. Obtusin, although exhibiting a relatively higher CDocker energy (−24.5937 kcal/mol), has a significantly lower CDocker interaction energy of −44.5619 kcal/mol. This discrepancy suggests that while its overall binding stability may be weaker, it still forms strong interactions within the NIK active site, warranting further consideration.

1-3-tribromoacetone, xanthosine, and 3-indoleacetamide exhibit moderate binding affinities, with CDocker energy scores ranging from −23.8405 to −23.0780 kcal/mol. Their interaction energies vary, with xanthosine (−45.7498 kcal/mol) showing particularly strong interactions, which could make it a valuable candidate despite its higher docking energy.

Lower-ranked compounds, such as isoflavone, phenylacetamide, and tryptophol, demonstrate relatively higher CDocker energy values, indicating less favorable binding conformations. Their interaction energies are also lower, suggesting that they form weaker interactions with NIK. Finally, pentabromophenol and tubermycin B rank lowest in this list, with CDocker energies of −12.4608 and −12.4318 kcal/mol, respectively. While their interaction energies are moderate, their overall binding affinity appears weak, making them the least promising candidates for NIK inhibition based on these docking energy values.

#### Molecular Docking Interactions

The molecular docking interaction analysis of the top 10 marine compounds against NIK was carried out, revealing a variety of binding modes, including direct hydrogen bonding and hydrophobic interactions. In this study, we only focused on the strong interaction, hydrogen bond interaction. No salt bridges were observed during the analysis (Table 2). Santacruzamate A, the top-ranked compound based on CDocker energy, interacts with the cysteine residue Cys533 at a distance of 2.34 Å. Actinonine, the second-best docked compound, interacts with multiple residues, including Leu472 and Arg408. It forms hydrogen bonds at distances of 2.40 Å and 2.20 Å with Leu472, and a particularly strong interaction with Arg408 at 2.07 Å. These shorter binding distances imply a tight interaction, which might enhance its inhibitory potential.

**Figure 2:**
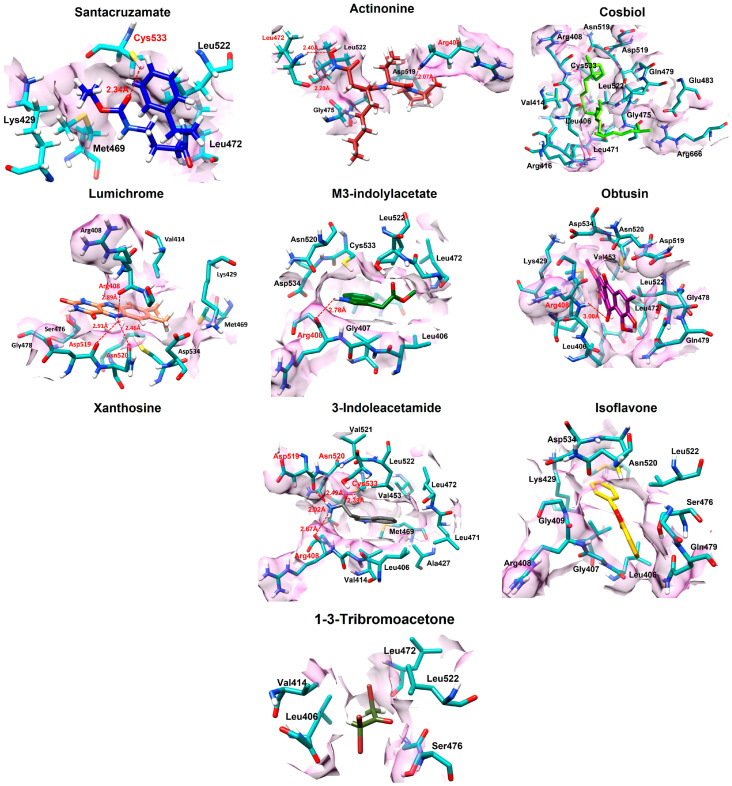
The interactions of the screened compounds are manifested in this figure. Each compound is colored differently, while the active site amino acid residues and their surfaces are colored light sea green and orchid, respectively. The hydrogen bonds, bonding distances, and identities of interacting amino acids are colored red, while the identities of other interacting amino acid residues are colored black.

Lumichrome forms multiple hydrogen bonds with Arg408, Asp519, and Asn520, with binding distances of 2.89 Å, 2.91 Å, and 2.46 Å, respectively. These interactions, though slightly longer, indicate moderate bonding strength. Arg408 plays a recurrent role in interactions across several compounds, suggesting that it may be a key residue in stabilizing ligands within the NIK binding site. M3-indolylacetate and obtusin also interact with Arg408, forming a bond at a distance of 2.48 Å and 3.00 Å, respectively (Figure 2). The closeness of the bond of m3-indolylacetate suggests a favorable interaction, while a relatively long distance of obtusin (3.00 Å) indicates a weaker interaction. The single residue interaction may limit the overall binding strength.

Xanthosine demonstrates a more complex interaction pattern, engaging with several residues, including Ser476, Asn520, Arg408, Glu470, and Leu472. The binding distances range from 1.90 Å to 2.75 Å, with particularly strong interactions at 1.93 Å and 2.19 Å with Asn520 and Ser476, respectively. These close interactions suggest a high level of binding stability. 3-indoleacetamide also exhibits multiple interactions, notably with Arg408, Asp519, Asn520, and Cys533. The binding distances are all relatively short, ranging from 2.02 Å to 2.67 Å, suggesting good interaction strength with the protein. The interactions with key residues like Arg408 and Cys533 may enhance its binding stability.

Cosbiol primarily manifests hydrophobic interactions rather than direct hydrogen bonds. This type of interaction suggests that cosbiol binds to a more hydrophobic pocket within NIK, stabilizing its position without forming hydrogen bond interactions. Though effective, hydrophobic interactions generally provide less binding strength compared to direct bonding. Similarly, 1-3-tribromoacetone and isoflavone relies solely on hydrophobic interactions, suggesting it binds less tightly to the active site compared to compounds with hydrogen bond formation.

### 3.4. Molecular Dynamic Simulation

To assess the stability of the screened compounds against NIK, the top 10 docked complexes were subjected to 100 ns MD simulations using GROMACS for further confirmation of the dynamic assessments, to accurately evaluate the potential of these compounds as NIK inhibitors.

#### 3.4.1. Root Mean Square Analysis

The Root Mean Square (RMSD) analysis of the simulated compounds provides valuable insights into their stability over a 100 ns molecular dynamics (MD) simulation. Santacruzamate A, lumichrome, xanthosine, and 3-indoleacetamide, all of which performed reasonably well in docking studies, demonstrated highly stable and similar fluctuating behavior throughout the simulation. Their consistent RMSD values over time suggest strong binding stability, corroborating their favorable docking scores. Santacruzamate A, for example, ranked first in molecular docking, with the lowest CDocker energy, and its stability in the MD simulation reinforces its potential as an effective NIK inhibitor.

**Figure 3:**
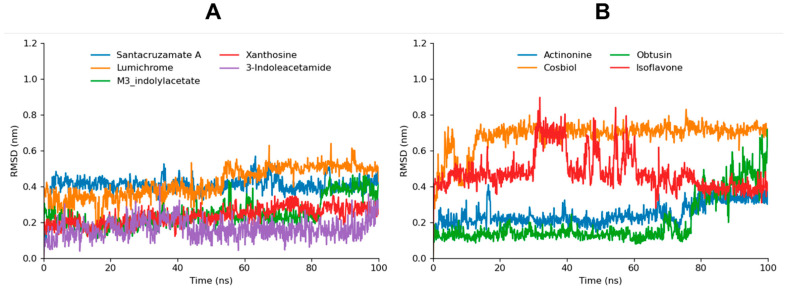
The RMSD graphs of santacruzamate A, xanthosine, lumichrome, 3-indoleacetamide, and m3-indolylacetate are depicted in graph (**A**), while RMSD graphs of actinonine, obtusin, isoflavone, and cosbiol are shown in graph (**B**).

Actinonine, which ranked second in molecular docking based on its strong interactions with NIK, showed a slight frameshift at 75 ns during the simulation. Similarly, m3-indolylacetate, ranked fifth in molecular docking, exhibited a frameshift at 80 ns (Figure 3). While its docking score suggested high binding affinity, the RMSD shift indicates a possible conformational change.

Cosbiol, which ranked third in molecular docking, manifested the highest RMSD values during the simulation. Although the RMSD bar is very consistent and stable, the value remained high. These results correlate with its docking, where it displayed only hydrophobic interactions with NIK. Isoflavone, which also primarily formed hydrophobic interactions in docking studies, showed high fluctuations from 30 ns to 80 ns in the simulation. However, after 80 ns, its RMSD values stabilized, indicating that despite initial instability, it may achieve a more stable binding conformation after structural optimization.

Obtusin remained relatively stable until 75 ns, after which its RMSD values increased significantly, indicating that it undergoes fluctuations towards the end of the simulation. Despite its moderate performance in molecular docking, the increased fluctuations in the RMSD analysis suggest that its binding to NIK may be less stable in a dynamic environment, potentially reducing its effectiveness. 1-3-tribromoacetone manifests the highest fluctuations, demonstrating nearly no potential as an NIK inhibitor in our study (Supplementary data Figure S1).

Overall, the RMSD analysis reveals compounds like santacruzamate A, actinonine, m3-indolylacetate, xanthosine, 3-indoleacetamide, and lumichrome emerging as the stable and effective inhibitors of NIK. Cosbiol, despite its favorable docking score, shows high RMSD values, emphasizing the importance of MD simulations in validating docking predictions. Isoflavone and obtusin exhibit varying degrees of instability, further confirming the need for dynamic assessments to accurately evaluate the potential of these compounds as NIK inhibitors.

#### 3.4.2. Hydrogen Bonds

The hydrogen bond plot analysis of the simulated compounds offers further insight into their binding interactions. Santacruzamate A, xanthosine, lumichrome, and actinonine each demonstrated the formation of nearly two actual hydrogen bonds during the 100 ns MD simulation, along with several potential hydrogen bonds. Among these, santacruzamate A exhibited the highest number of actual hydrogen bonds, reinforcing its strong and stable binding performance as observed in the RMSD analysis, where it remained highly stable throughout the simulation.

Xanthosine, lumichrome, and actinonine, while showing similar numbers of actual hydrogen bonds, also manifested a high number of potential hydrogen bonds. This additional potential for hydrogen bonding suggests flexibility in their binding interactions, allowing these compounds to adapt to the dynamic changes during the simulation. In the RMSD analysis, xanthosine remained highly stable, consistent with its hydrogen bond profile, which further solidifies its position as a promising NIK inhibitor. Lumichrome and actinonine, despite slight fluctuations noted in the RMSD data, maintained significant hydrogen bond interactions, particularly in actinonine, where its strong hydrogen bond network helped stabilize the compound.

M3-indolylacetate and 3-indoleacetamide both formed one actual hydrogen bond during the 100 ns trajectory, but m3-indolylacetate showed a higher number of potential hydrogen bonds, including three stable potential hydrogen bonds. This finding aligns with the RMSD, suggesting that its hydrogen bond interactions, though fewer in number, may play a role in maintaining some level of stability throughout the simulation. In comparison, 3-indoleacetamide formed fewer stable potential hydrogen bonds, and while its RMSD analysis indicated good stability, the lower hydrogen bonding could suggest that other interaction types contribute to its overall stability within the binding site.

Cosbiol, obtusin, isoflavone, and 1-3-tribromoacetone showed the lowest hydrogen bond interactions, with minimal actual or potential hydrogen bonds formed during the simulation. This result correlates with the higher RMSD fluctuations seen in cosbiol and obtusin. The lack of significant hydrogen bonding for these compounds suggests weaker interactions with NIK, aligning with their moderate to poor stability in the RMSD analysis, and reflecting a lower potential for effective inhibition.

**Figure 4:**
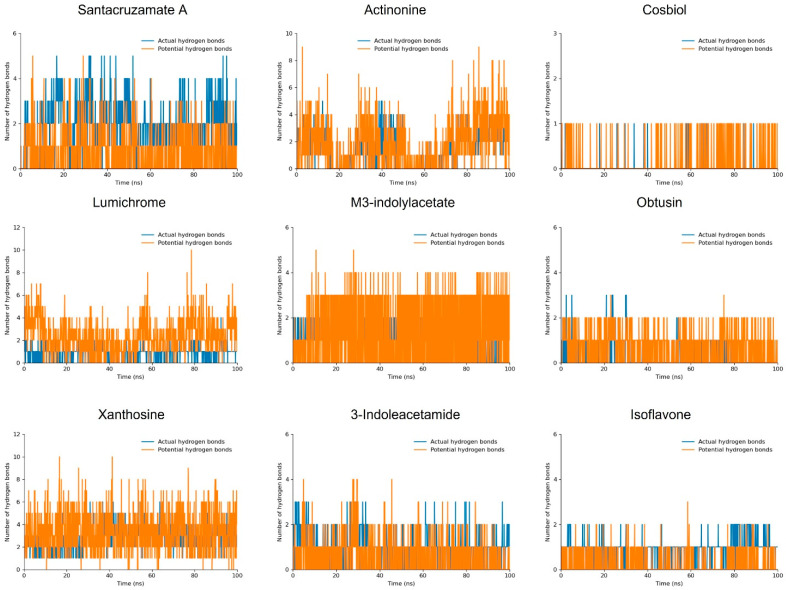
The hydrogen bond plot shows two types of bar lines: the blue represents actual hydrogen bonds, while the orange depicts potential hydrogen bonds.

Overall, compounds like santacruzamate A, xanthosine, lumichrome, and actinonine, which show high numbers of actual and potential hydrogen bonds, exhibit good stability during the MD simulation, as evidenced by their robust hydrogen bond networks. On the other hand, compounds such as cosbiol, obtusin, isoflavone, and 1-3-tribromoacetone, which formed fewer hydrogen bonds, exhibited moderate instability (Supplementary data Figure S2) (Figure 4).

#### 3.4.3. MD Interaction Energy

**Figure 5:**
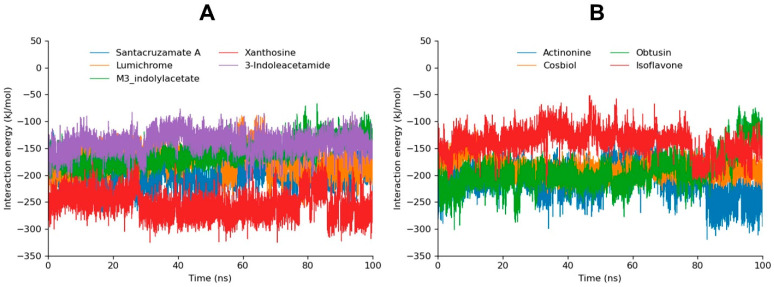
The MD interaction energy graphs of santacruzamate A, xanthosine, lumichrome, 3-indoleacetamide, and m3-indolylacetate are depicted in graph (**A**). Moreover, the interaction energy graphs of actinonine, obtusin, isoflavone, and cosbiol are shown in graph (**B**).

The binding free energy analysis of the simulated compounds provides an in-depth look into the strength and stability of their interactions with NIK, further expanding on the results from RMSD and hydrogen bond analyses. The interaction energy was calculated from 0 ns to 100 ns, depicting the whole trajectory (Figure 5).

Santacruzamate A and xanthosine, with total binding free energies of −299.5937 kJ/mol and −254.726 kJ/mol, respectively, stand out as the most potent inhibitors, showing the strongest binding affinity. These exceptionally high binding energies correlate with their top performance in both the RMSD and hydrogen bond analyses, where they demonstrated the highest number of actual and potential hydrogen bonds and remained highly stable throughout the 100 ns MD simulation. The strong energy values reflect the compounds’ robust interactions with NIK, underscoring their potential as highly effective inhibitors.

Actinonine and m3-indolylacetate, with total binding energies of −209.6835 kJ/mol and −201.9300 kJ/mol, respectively, also performed well, ranking 2nd and 5th in the docking analysis. The high interaction energy seen here aligns with its ability to form multiple hydrogen bonds. Despite slight frameshifts at 75 ns and 80 nss respectively, the overall binding energy remains strong, indicating that actinonine maintains significant interactions with NIK, which contribute to its stable binding profile.

Cosbiol, despite ranking third in molecular docking, has a much weaker total binding free energy of −130.1227 kJ/mol. Lumichrome, which demonstrated good stability in the hydrogen bond analysis, showed a more moderate binding affinity compared to the top compounds, with a total binding energy of −171.0612 kJ/mol (Table 3). Obtusin, with a total binding energy of −193.8964 kJ/mol, exhibited strong binding affinity, showing it was bound potently before the fluctuations seen in the RMSD at 75 ns, warranting the structural optimization of this compound.

3-indoleacetamide and isoflavone, with total binding energies of −140.5801 kJ/mol and −138.4748 kJ/mol, respectively, showed moderate interaction strength, which aligns with their RMSD and hydrogen bond profiles, where they exhibited fewer hydrogen bonds than other top compounds. The lower binding energies suggest a weaker binding conformation, reflecting overall low compatibility, as seen across the various analyses. 1-3-tribromoacetone manifested the lowest MD interaction, corresponding to RMSD results (Supplementary data Figure S3).

### 3.5. Binding Free Energy Analysis

gmxMMPBSA tool was employed for binding free energy calculation of the top six compounds that depicted high stability profiles in MD simulation analysis. The free energy calculation plays a critical role in molecular dynamics studies by providing a detailed estimation of binding free energies between a ligand and a target protein. It allows the evaluation of the energetics of molecular interactions in a biologically relevant environment. This analysis is essential for understanding the thermodynamic stability of ligand–receptor complexes. By providing a more accurate prediction of binding affinities, gmxMMPBSA helps in identifying potent inhibitors and optimizing lead compounds.

Santacruzamate A, with a ΔG of −24.29 kcal/mol, demonstrates the most favorable binding free energy. This strong binding affinity is consistent with the MD simulation analysis, where santacruzamate A exhibited remarkable stability throughout the 100 ns. The combination of strong free energy, stable RMSD behavior, and significant hydrogen bonding interactions positions santacruzamate A as a leading candidate for NIK inhibition. Actinonine, with a ΔG of −19.76 kcal/mol, also exhibits a strong binding free energy, second only to santacruzamate A. The stable RMSD analysis and the hydrogen bond analysis highlighted its ability to form multiple actual and potential hydrogen bonds. These interactions contribute to its favorable binding energy, making actinonine a reliable inhibitor with strong and stable interactions with NIK.

M3-indolylacetate and xanthosine, with ΔGs of −15.3 kcal/mol and −17.13 kcal/mol and with standard deviations of 3.69 and 3.88, respectively, demonstrate moderately strong binding free energy, aligning with their performance in the RMSD analysis (Table 4). Xanthosine and m3-indolylacetate also formed stable potential hydrogen bonds throughout the simulation. The free energy results confirm that m3-indolylacetate has a solid interaction with NIK, with relatively stable dynamics contributing to its overall binding affinity.

Lumichrome and 3-indoleacetamide, with ΔGs of −9.05 kcal/mol and −11.9 kcal/mol and with standard deviations of 3.78 and 3.87, respectively, have a comparatively weaker free energy than santacruzamate A and actinonine. In the RMSD analysis, lumichrome displayed stable behavior, and the hydrogen bond analysis showed it formed a moderate number of hydrogen bonds. 3-indoleacetamide also maintained stable interactions in RMSD analysis, though it formed fewer hydrogen bonds than other top compounds. Despite its stability in these aspects, the lower free energy indicates weaker binding strength, suggesting that while lumichrome and 3-indoleacetamide maintain stable interactions with NIK, their inhibitory potentials are less than the top compounds.

**Figure 6:**
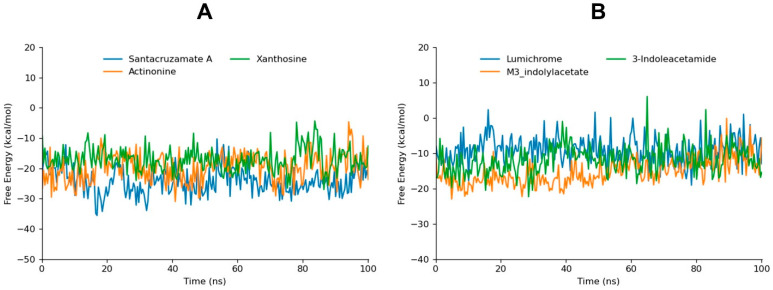
The gmxMMPBSA free energy graphs of santacruzamate A, xanthosine, and actinonine are depicted in graph (**A**). Furthermore, the gmxMMPBSA free energy graphs of m3-indolylacetate, lumichrome, and 3-indoleacetamide are shown in graph (**B**).

Overall, the free energy calculation data align with the findings from RMSD and hydrogen bond analyses, highlighting santacruzamate A, actinonine, and xanthosine as the most promising NIK inhibitors due to their strong binding energies, stable molecular dynamics, and robust hydrogen bonding interactions (Figure 6). Lumichrome, m3-indolylacetate, and 3-indoleacetamide, while showing reasonable binding affinities, exhibit weaker free energies compared to the top compounds, reflecting their more moderate stability and interaction profiles.

**Figure 7:**
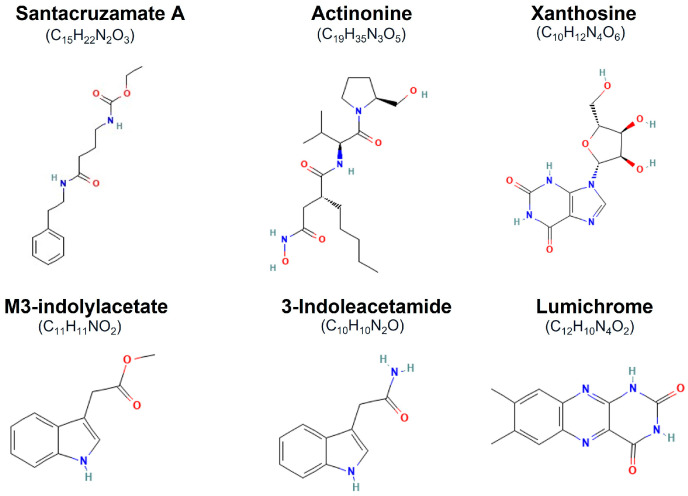
The 2D representation of the top 6 compounds subjected to free energy calculation.

The diverse activities of the top listed compounds highlight the potential applications in various fields, from agriculture to medicine, though some require further research to fully understand their mechanisms and therapeutic potential. The 2D representation of those compounds are manifested in Figure 7. Among the top listed compounds, santacruzamate A was initially reported as a potent histone deacetylase 2 (HDAC2) inhibitor, Some santacruzamate A analogues have shown anti-proliferative effects on cancer cells and immune-modulating properties in subsequent studies [38]. Actinonine, a natural antibiotic, inhibits peptide deformylase in bacteria and human mitochondria, demonstrating antibacterial, antitumor, and anti-angiogenic properties [39,40]. Xanthosine, a nucleoside involved in purine metabolism, has been studied for increasing milk production in dairy cows and potential neuroprotective effects [41]. M3-indolylacetate and 3-indoleacetamide are plant hormones regulating growth and development, with studies indicating potential anticancer and neuroprotective properties [42]. Lastly, lumichrome, a riboflavin photodegradation product, stimulates plant growth, exhibits antimicrobial properties, and shows potential as an anticancer agent [43,44].

## 4. Conclusions

NF-κB-inducing kinase (NIK) plays a critical role in driving inflammation, autoimmune disorders, and cancer progression. By focusing on marine-derived compounds, we explored an untapped reservoir of natural bioactive molecules capable of inhibiting NIK. Through molecular docking, molecular dynamics (MD) simulations, and binding energy calculations, we identified several promising candidates, with santacruzamate A, actinonine, and xanthosine emerging as the top performers. These compounds exhibited strong binding affinities, stable interactions, and favorable free energy profiles, suggesting their potential to effectively inhibit NIK activity.

Among them, santacruzamate A stood out due to its consistently robust performance across various analytical methods, positioning it as a leading candidate for further investigation. The stability of its interactions and high binding energy highlight its potential as a therapeutic agent targeting NIK. These findings not only reinforce the therapeutic relevance of targeting NIK in diseases such as inflammation and cancer but also demonstrate the value of marine compounds as a rich source of novel inhibitors. This study paves the way for future research into natural anti-inflammatory therapies, leveraging the unique properties of marine compounds to disrupt key signaling pathways involved in disease progression.

## Figures and Tables

**Table 1:** The docking energy values of the top 15 compounds, ranked considering the overall CDocker energy values.

Sr No	Compounds	Molecular Formula	CDocker Energy (kcal/mol)	CDocker Interaction Energy (kcal/mol)
1	Santacruzamate A	C_15_H_22_N_2_O_3_	−49.0692	−44.9589
2	Actinonine	C_19_H_35_N_3_O_5_	−45.5211	−52.1040
3	Cosbiol	C_30_H_62_	−34.8735	−49.9577
4	Lumichrome	C_12_H_10_N_4_O_2_	−31.5584	−35.1686
5	M3-indolylacetate	C_11_H_11_NO_2_	−26.8642	−29.1885
6	Obtusin	C_18_H_16_O_7_	−24.5937	−44.5619
7	1-3-Tribromoacetone	C_3_H_3_Br_3_O	−23.8405	−22.2132
8	Xanthosine	C_10_H_12_N_4_O_6_	−23.1161	−45.7498
9	3-Indoleacetamide	C_10_H_10_N_2_O	−23.0780	−26.7549
10	Isoflavone	C_15_H_10_O_2_	−20.8883	−28.6739
11	Phenylacetamide	C_8_H_9_NO	−20.5446	−22.1003
12	2-6-Dibromophenol	C_7_H_3_Br_2_NO	−20.3419	−23.9437
13	Tryptophol	C_10_H_11_NO	−20.0190	−25.7352
14	Pentabromophenol	C_6_HBr_5_O	−12.4608	−35.6949
15	Tubermycin B	C_13_H_8_N_2_O_2_	−12.4318	−32.6023

**Table 2:** The interacting amino acids and the binding distance of the top 10 screened compounds against the active site amino acid residues of NIK.

Compounds	Interacting Residues	Binding Distances
Santacruzamate A	Cys533	2.34 Å
Actinonine	Leu472 Arg408	2.40 Å, 2.20 Å 2.07 Å
Cosbiol	Hydrophobic Interactions	
Lumichrome	Arg408 Asp519 Asn520	2.89 Å 2.91 Å 2.46 Å
M3-indolylacetate	Arg408	2.48 Å
Obtusin	Arg408	3.00 Å
1-3-Tribromoacetone	Hydrophobic Interactions	
Xanthosine	Ser476 Asn520 Arg408 Glu470 Leu472	2.19 Å 1.93 Å 2.24 Å 2.30 Å 2.75 Å, 1.90 Å
3-Indoleacetamide	Arg408 Asp519 Asn520 Cys533	2.67 Å 2.02 Å 2.49 Å 2.39 Å
Isoflavone	Hydrophobic Interactions	

**Table 3:** The MD interaction energy of the simulated compounds was calculated during the 100 ns MD simulation.

Sr No	Compound	Interaction Energy (kJ/mol)		
		Coul-SR	LJ-SR	Total Energy
1	Santacruzamate A	−155.8117	−143.7820	−299.5937
2	Xanthosine	−126.1830	−128.5430	−254.7260
3	Actinonine	−61.7505	−147.9330	−209.6835
4	M3-indolylacetate	−81.5560	−120.3740	−201.9300
5	Obtusin	−50.1384	−143.7580	−193.8964
6	Lumichrome	−54.9542	−116.1070	−171.0612
7	3-Indoleacetamide	−39.6131	−100.9670	−140.5801
8	Isoflavone	−41.9749	−96.4999	−138.4748
9	Cosbiol	−0.2207	−129.9020	−130.1227
10	1-3-Tribromoacetone	−4.1828	−1.2765	−5.4593

**Table 4:** The calculated gmxMMPBSA free energy of the top six screened compounds.

Sr	Compounds	ΔG_(TOTAL)_	Standard Deviation
1	Santacruzamate A	−24.29	4.26
2	Actinonine	−19.76	4.07
3	Xanthosine	−17.13	3.88
4	M3-indolylacetate	−15.3	3.69
5	3-Indoleacetamide	−11.9	3.87
6	Lumichrome	−9.05	3.78

## Data Availability

The data that support the findings of this study are available from the corresponding author upon reasonable request.

## Supplementary Materials

The following supporting information can be downloaded at: https://www.mdpi.com/article/10.3390/biom14121490/s1, Table S1: All Molecular docking results of the marine sourced compound library; Figure S1: The RMSD graph of highly fluctuating 1-3-Tribromoacetone; Figure S2: The hydrogen bond plot of 1-3-Tribromoacetone; Figure S3: The interaction energy plot of 1-3-Tribromoacetone.
