# Supplementary material for: Identification of Marine Compounds Inhibiting NF-κBInducing Kinase Through Molecular Docking and Molecular Dynamics Simulations
Source: Biomolecules. 2024 Nov 22;14(12):1490. doi: 10.3390/biom14121490 (PMC11673129; doi:10.3390/biom14121490)
Supplement: Supplementary file 1 [file biomolecules-14-01490-s001.zip › biomolecules-3309434-supplementary.pdf]

Supplementary Data

Identification of Potent Marine Compounds Targeting NF-κB Inducing Kinase (NIK) Through Molecular Docking and Dynamics Simulations

Muhammad Yasir[1], Jinyoung Park[1], Eun-Taek Han[2], Jin-Hee Han[2], Won Sun Park[3], Wanjoo Chun[1,*]

[1]Department of Pharmacology, Kangwon National University School of Medicine, Chuncheon, 24341, Republic of Korea;

[2]Department of Medical Environmental Biology and Tropical Medicine, Kangwon National University School of Medicine, Chuncheon, 24341, Republic of Korea;

[3]Department of Physiology, Kangwon National University School of Medicine, Chuncheon, 24341, Republic of Korea;

Corresponding author: Dr. Wanjoo Chun, Department of Pharmacology Kangwon National University School of Medicine, Kangwon National University, Email: wchun@kangwon.ac.kr, Phone: +82-33-250-8853.

Table S1. All Molecular docking results of the marine sourced compound library.

Sr No	Compounds	CDocker Energy (kcal/mol)	CDocker Interaction Energy (kcal/mol)
1	Santacruzamate_A	-49.0692	-44.9589
2	Actinonine	-45.5211	-52.1040
3	Cosbiol	-34.8735	-49.9577
4	Lumichrome	-31.5584	-35.1686
5	Methyl_3-indolylacetate	-26.8642	-29.1885
6	Obtusin	-24.5937	-44.5619
7	1_3-Tribromoacetone	-23.8405	-22.2132
8	Xanthosine	-23.1161	-45.7498
9	3-Indoleacetamide	-23.0780	-26.7549
10	Isoflavone	-20.8883	-28.6739
11	Phenylacetamide	-20.5446	-22.1003
12	2_6-DIBROMOPHENOL	-20.3419	-23.9437
13	Tryptophol	-20.0190	-25.7352
14	Pentabromophenol	-12.4608	-35.6949
15	Tubermycin_B	-12.4318	-32.6023
16	N6-Dimethyladenosine	-12.1854	-40.2933
17	Jaspamycin	-11.2678	-39.5537
18	Dehydroabietate	-11.0756	-37.7845
19	Coixol	-10.9186	-22.5825
20	3-Methylcytidine	-9.3297	-38.2710
21	Adenosine	-7.2456	-33.7036
22	Isoquinoline	-5.6268	-19.9226
23	Ellipticine	-5.2431	-37.6701
24	Citronellol	-3.3055	-28.7289
25	Deoxylapachol	0.4641	-32.1425
26	Isatin	1.0611	-22.8708
27	Tadeonal	13.2932	-24.7528
28	Nerol	17.4609	-27.5050
29	Lemonol	17.9545	-27.9811
30	Furanodiene	20.2909	-29.7135
31	Dieckol	22.2224	-57.4370
32	Picrotin	22.6080	-26.7207
33	Arcyriaflavin_A	23.1142	-40.6062
34	Vetivazulen	27.7706	-31.3604
35	Isopteropodine	30.0881	-36.8321

36	Picrotoxinin	40.9511	-22.9422
37	Smilagenin	73.1898	-29.7475
38	Scytonemin	74.1289	-50.6498
39	Staurosporin	111.6120	-46.4818
40	Peroxyergosterol	134.2430	-22.4230

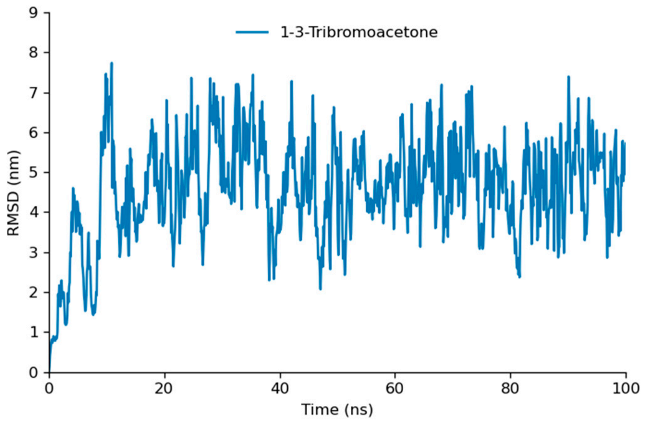

Figure S1. The RMSD graph of highly fluctuating 1-3-Tribromoacetone.

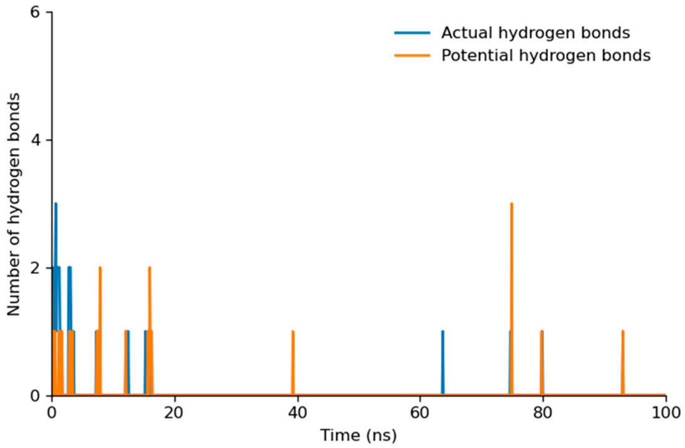

Figure S2. The hydrogen bond plot of 1-3-Tribromoacetone.

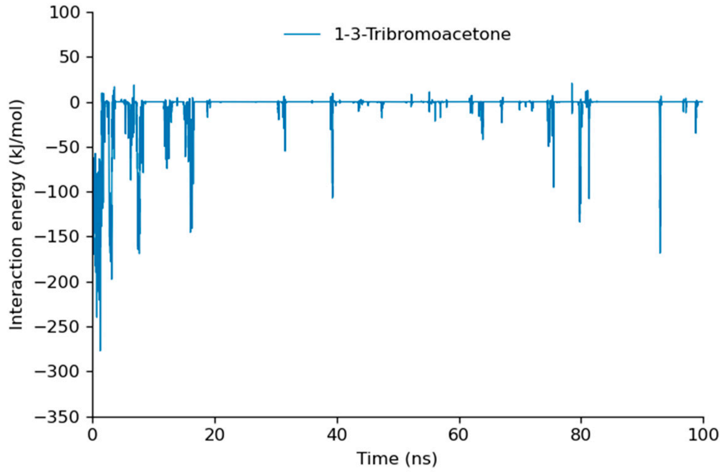

Figure S3. The interaction energy plot of 1-3-Tribromoacetone.
